# Child maltreatment and health service use: findings of the Australian Child Maltreatment Study

**DOI:** 10.5694/mja2.51892

**Published:** 2023-04-02

**Authors:** Rosana Pacella, Alexandra Nation, Ben Mathews, James G Scott, Daryl J Higgins, Divna M Haslam, Michael P Dunne, David Finkelhor, Franziska Meinck, Holly E Erskine, Hannah J Thomas, Eva Malacova, David M Lawrence, Claire Monks

**Affiliations:** ^1^ Institute for Lifecourse Development University of Greenwich London United Kingdom; ^2^ Queensland University of Technology Brisbane QLD; ^3^ Bloomberg School of Public Health Johns Hopkins University Baltimore MD United States of America; ^4^ Child Health Research Centre the University of Queensland Brisbane QLD; ^5^ QIMR Berghofer Medical Research Institute Brisbane QLD; ^6^ Institute of Child Protection Studies, Australian Catholic University Melbourne VIC; ^7^ The University of Queensland Brisbane QLD; ^8^ Institute for Community Health Research Hue University Hue City Vietnam; ^9^ Crimes against Children Research Center University of New Hampshire Durham NH United States of America; ^10^ University of Edinburgh Edinburgh United Kingdom; ^11^ North‐West University Potchefstroom South Africa; ^12^ Queensland Centre for Mental Health Research Brisbane QLD; ^13^ Curtin University Perth WA

**Keywords:** Child welfare, Community health services, Cost of illness, Health services

## Abstract

**Objectives:**

To examine associations between child maltreatment and health service use, both overall, by type and by the number of types of maltreatment reported.

**Design, setting:**

Cross‐sectional, retrospective survey using the Juvenile Victimization Questionnaire‐R2: Adapted Version (Australian Child Maltreatment Study); computer‐assisted mobile telephone interviews using random digit dialling, Australia, 9 April – 11 October 2021.

**Participants:**

Australians aged 16 years or more. The target sample size was 8500 respondents: 3500 people aged 16–24 years and 1000 respondents each from the five age groups (25–34, 35–44, 45–54, 55–64, 65 years or more).

**Main outcome measures:**

Self‐reported health service use during the past twelve months: hospital admissions, length of stay, and reasons for admission; and numbers of consultations with health care professionals, overall and by type. Associations between maltreatment and health service use are reported as odds ratios adjusted for age group, gender, socio‐economic status, financial hardship (childhood and current), and geographic remoteness.

**Results:**

A total of 8503 participants completed the survey. Respondents who had experienced child maltreatment were significantly more likely than those who had not to report a hospital admission during the preceding twelve months (adjusted odds ratio [aOR], 1.39; 95% confidence interval [CI], 1.16–1.66), particularly admission with a mental disorder (aOR, 2.4; 95% CI, 1.03–5.6). The likelihood of six or more visits to general practitioners (aOR, 2.37; 95% CI, 1.87–3.02) or of a consultation with a mental health nurse (aOR, 2.67; 95% CI, 1.75–4.06), psychologist (aOR, 2.40; 95% CI, 2.00–2.88), or psychiatrist (aOR, 3.02; 95% CI, 2.25–4.04) were each higher for people who reported maltreatment during childhood. People who reported three or more maltreatment types were generally most likely to report greater health service use.

**Conclusions:**

Child maltreatment has a major impact on health service use. Early, targeted interventions are vital, not only for supporting children directly, but also for their longer term wellbeing and reducing their health system use throughout life.

**The known:** Adverse health outcomes associated with child maltreatment lead to greater health service use throughout life.

**The new:** Our survey found that child maltreatment in Australia, particularly the experience of multiple types of maltreatment, is associated with increased likelihood of hospital admission with mental disorders, and higher numbers of consultations with health care professionals of various types.

**The implications:** Our findings confirm the impact of maltreatment during childhood on health service use. Alongside effective prevention strategies, integrated physical and psychological health care is needed to reduce the harms caused by maltreatment and to improve long term health.

The Australian Child Maltreatment Study (ACMS) provides the first national estimates of the prevalence of child maltreatment (physical abuse, sexual abuse, emotional abuse, neglect, and exposure to domestic violence) in Australia.^1^, ^2^ As reported in this Supplement, 62.2% of survey participants reported they had experienced at least one of the five types of maltreatment before the age of 18 years, 39.4% reported more than one type of maltreatment, and 23.3% reported three or more types of maltreatment.^2^

Causal relationships have been established between child maltreatment and mental and physical health outcomes across life,^3^, ^4^, ^5^, ^6^ and concomitant increases in health service use have been documented.^7^, ^8^ A prospective cohort study in the United States found that people with histories of maltreatment during childhood were more likely to report using mental health and social services as adults;^9^ another large study found increased health care use by women who experienced physical or sexual abuse, and annual health care costs were 36% higher for those who experienced both maltreatment types.^10^ A Canadian study found that self‐reported annual health care costs were 94% higher for women who experienced physical and sexual abuse during childhood.^11^ A birth cohort study in South Australia found that emergency department visit rates, particularly visits during adolescence and early adulthood related to self‐harm, substance use, and mental health, were higher for people who reported earlier contact with child protection services; people who had been maltreated sought help related to substance use or mental illness 3–15 times as frequently as people who had not been maltreated.^12^

Notwithstanding data limitations and uncertainty about its exact magnitude, the economic burden of child maltreatment is great.^13^, ^14^, ^15^, ^16^ Given the implications for governments of increased demands on limited health care resources, understanding patterns of health service use by people maltreated as children is important for reliable estimates of their health care use across life.

In the ACMS, child maltreatment was associated with greater risk of mental disorders,^17^ self‐harm, suicide attempts, and health risk behaviours and conditions such as cannabis dependence, obesity, smoking, and alcohol binge drinking that are major risk factors for non‐communicable diseases.^18^ We analysed ACMS data to assess associations between child maltreatment and health service use, both overall, by type, and by whether one or multiple types of maltreatment were experienced.

## Methods

The ACMS is a cross‐sectional survey study of people in Australia aged 16 years or more during 9 April – 11 October 2021 about their childhood and health. The methodology and definitions of child maltreatment are described elsewhere.^19^, ^20^ In brief, participants were recruited using a mobile phone sampling frame and random digit dialling methodology. The target survey respondent number was 8500: 3500 people aged 16–24 years (oversampled) and 1000 in each of the age groups 25–34, 35–44, 45–54, 55–64, and 65 years or more. To ensure that the sample was representative of the population, survey data were weighted by age group, gender, Indigenous status, country of birth (Australia or overseas), highest educational level, and residential socio‐economic status (Socio‐Economic Indexes for Areas [SEIFA] Index of Relative Socio‐economic Advantage and Disadvantage).^19^

### Measures

Child maltreatment was assessed with the Juvenile Victimization Questionnaire‐R2: Adapted Version (Australian Child Maltreatment Study).^19^, ^20^ The five types of child maltreatment (physical abuse, sexual abuse, emotional abuse, neglect, and exposure to domestic violence) were assessed by sixteen survey items. We assessed self‐reported health service use with items from the 2007 National Survey of Mental Health and Wellbeing health service utilisation module,^21^ with minor modifications required for the ACMS, including health service use related to coronavirus disease 2019 (COVID‐19). The selected items included hospital use (“In the past 12 months, how many times were you admitted overnight to any hospital (excluding childbirth)?”: 0, 1 or more times) and hospital length of stay (“How many nights in total did you stay overnight in hospital?”). In addition, we collected information on the reason for overnight hospital admission with a list of nine mental health and eleven physical health conditions (including “other”; multiple reasons could be given).

Participants also reported consultations with health care professionals (“How many times did you see each of the following health professionals in the last 12 months?”; 0, 1–5, 6–11, 12–23, 24 or more times), including general practitioners, psychologists, psychiatrists, mental health nurses, allied health professionals (social workers, counsellors, physiotherapists, occupational therapists), and specialist physicians. We asked about use during the preceding twelve months to minimise recall bias, and because we assumed participants would remember reasons for hospitalisation during this time period. We did not ask about reasons for health professional consultations as there would often have been several reasons, and to minimise participant burden and recall bias.

Financial hardship during childhood was assessed with the question: “How often did your family experience economic hardship such as finding it difficult to provide food, medical care, or other basic necessities?” Childhood financial hardship was recorded for participants who responded “somewhat often” or “very often”.

Based on residential postcode, we assessed socio‐economic status with the SEIFA Index of Relative Socio‐economic Disadvantage (IRSD; by quintile),^22^ and remoteness with the Australian Statistical Geography Standard Remoteness Structure (major cities, inner regional, outer regional, remote).^23^ Current financial hardship was assessed with the question: “In the past 12 months, has there been a time when your household could not meet its essential expenses?” (yes/no response option).

### Statistical analysis

For each question on child maltreatment and health service use, fewer than 1% of participants declined to respond. We conservatively chose to treat missing data as non‐endorsements (negative responses).

Associations between reported child maltreatment and health service use were examined in three survey‐weighted logistic regression models. In the first model, adjusted odds ratios (aORs) and 95% confidence intervals (CIs) were estimated for health service use by child maltreatment (any *v* none); in the second, for health service use by each of the five types of child maltreatment, fitted simultaneously as independent binary (yes/no) variables, enabling associations between health service use and each maltreatment type to be adjusted for experiences of other types of maltreatment; and in the third model, for health service use by number of types of maltreatment (one, two, or three or more types). Two levels of adjustment for other factors were applied to each model: partial, which took into account basic demographic characteristics (age group, gender) and fully adjusted, which also took geographic and socio‐economic factors into account (socio‐economic status quintile, financial hardship during childhood, current financial stress, and geographic remoteness). The statistical significance of differences in survey‐weighted median hospital length of stay between participants who reported or did not report maltreatment during childhood was assessed in Mann–Whitney *U* tests.

All analyses were conducted in SAS 9.4. Each analysis was independently checked by two co‐authors, in random spot checks of SAS code and checking of analysis results in SPSS 28.

### Ethics approval

The study was approved by the Queensland University of Technology Human Research Ethics Committee (1900000477).

## Results

A total of 8503 Australian residents aged 16 years or more completed the ACMS survey, of whom 1218 (survey‐weighted proportion, 15.4%; 95% CI, 14.4–16.3%) had been admitted to hospital overnight at least once during the past year (857 of 5280 people who reported maltreatment [16.9%; 95% CI, 15.6–18.3%]; 361 of 3223 who reported no maltreatment [12.7%; 95% CI, 11.2–14.3%]) (Box 1). The survey‐weighted median length of stay for each group was three days (95% CI, 2.5–3.5 days). The survey‐weighted proportion for hospital admissions is slightly higher than both the unweighted value (14.3%) (Supporting Information, table 1) and the value reported for 2020–21 by the Australian Bureau of Statistics for people aged 15 years or more (12.5%).^24^

Box 1 **Likelihood of overnight hospital admission during the preceding twelve months (maltreatment reported *v* not reported), by maltreatment type**

**Table:** 

	Admissions		Adjusted odds ratio (95% CI)	
Maltreatment type	Numbers	Weighted proportion (95% CI)	Partial^*^	Full^†^
No maltreatment	361/3223	12.7% (11.2–14.3%)	1	1
Any maltreatment	857/5280	16.9% (15.6–18.3%)	1.52 (1.29–1.80)	1.39 (1.16–1.66)
Physical abuse	481/2623	18.5% (16.7–20.4%)	1.25 (1.04–1.51)	1.21 (1.00–1.46)
Sexual abuse	448/2348	19.2% (17.2–21.2%)	1.33 (1.10–1.59)	1.24 (1.03–1.50)
Emotional abuse	494/2743	18.3% (16.4–20.1%)	1.23 (1.00–1.51)	1.19 (0.97–1.47)
Neglect	146/759	17.5% (14.2–20.9%)	0.89 (0.68–1.18)	0.79 (0.59–1.07)
Exposure to domestic violence	576/3487	16.8% (15.2–18.4%)	1.11 (0.92–1.33)	1.08 (0.90–1.31)

CI = confidence interval.

* Adjusted for other maltreatment types (when applicable), age group, and sex.

† Adjusted for other maltreatment types (when applicable), age group, sex, socio‐economic status, financial hardship (childhood and current), and geographic remoteness.

### Any maltreatment during childhood

After adjusting for socio‐demographic characteristics, financial hardship (childhood and current), and geographic remoteness, people who reported maltreatment were more likely than those who did not to have had an overnight hospital admission during the past twelve months (aOR, 1.39; 95% CI, 1.16–1.66) (Box 1). Participants who reported any maltreatment were also more likely to report a mental disorder as the reason for hospitalisation (aOR, 2.4; 95% CI, 1.03–5.6); there was no significant difference in the odds of admission with depression (aOR, 3.3; 95% CI, 0.5–20), nor with physical health disorders or injury (Box 2).

Box 2 Likelihood of reasons for overnight hospital admission during the preceding twelve months (maltreatment reported *v* not reported)^*^

**Table:** 

	Adjusted odds ratio (95% CI)	
Reason for admission	Partial^†^	Full^‡^
Any mental disorder^§^	2.7 (1.1–6.5)	2.4 (1.03–5.6)
Anxiety	0.8 (0.3–2.3)	0.9 (0.4–2.2)
Depression	3.6 (0.6–21)	3.3 (0.5–20)
Drug‐related problems	3.7 (0.6–21)	2.2 (0.4–12)
Alcohol‐related problems	0.6 (0.2–2.1)	1.1 (0.4–3.7)
Suicide risk	3.8 (0.8–18)	1.4 (0.6–3.3)
Heart disease^¶^	1.7 (0.9–3.4)	1.3 (0.6–2.5)
Injury or results of injury	1.1 (0.7–1.7)	1.1 (0.7–1.6)
Asthma or chronic bronchitis	0.9 (0.2–3.4)	0.8 (0.2–3.1)
Diabetes	1.4 (0.3–6.8)	0.5 (0.1–2.2)
Cancer	1.0 (0.5–2.2)	1.1 (0.5–2.5)
Stroke	0.6 (0.2–1.7)	0.4 (0.1–0.97)

CI = confidence interval.

* A more comprehensive list of conditions, and the raw data underlying the adjusted odds ratios, are included in the Supporting Information, table 1.

† Adjusted for age group, and sex.

‡ Adjusted for age group, sex, socio‐economic status, financial hardship (childhood and current), and geographic remoteness.

§ Includes schizophrenia, anxiety, depression, eating disorders, bipolar disorder, personality disorders, and (from the “Other” category) mental health, post‐traumatic stress disorder, and panic attack.

¶ Includes heart attack and (from the “Other” category) heart disease, heart disease/anaemia, heart disease/pneumonia, heart disease/surgery, heart failure/arrhythmia, heart failure, heart problem, heart surgery, surgery/heart problem.

Participants who reported maltreatment were more likely to have visited general practitioners six or more times (aOR, 2.37; 95% CI, 1.87–3.02) and to have consulted a psychiatrist (aOR, 3.02; 95% CI, 2.25–4.04), a psychologist (aOR, 2.40; 95% CI, 2.00–2.88), or a mental health nurse (aOR, 2.67; 95% CI, 1.75–4.06). They were also more likely to have had at least one consultation with any medical specialist (aOR, 1.23; 95% CI, 1.08–1.40) and made six or more visits to allied health professionals (aOR, 1.60; 95% CI, 1.31–1.94) (Box 3).

Box 3 Likelihood of consultations with health care professionals during preceding twelve months (maltreatment reported *v* not reported)^*^

**Table:** 

	Adjusted odds ratio (95% CI)	
Consultation type	Partial^†^	Full^‡^
At least one visit to psychologist	2.69 (2.26–3.21)	2.40 (2.00–2.88)
At least one visit to psychiatrist	3.37 (2.52–4.49)	3.02 (2.25–4.04)
At least one visit to mental health nurse	3.16 (2.10–4.75)	2.67 (1.75–4.06)
At least one visit to any medical specialist	1.28 (1.13–1.45)	1.23 (1.08–1.40)
1–5 visits to general practitioners	1.33 (1.10–1.61)	1.37 (1.12–1.67)
6 or more visits to general practitioners	2.66 (2.12–3.35)	2.37 (1.87–3.02)
1–5 visits to allied health professionals	1.41 (1.20–1.64)	1.35 (1.14–1.59)
6 or more visits to allied health professionals	1.74 (1.44–2.09)	1.60 (1.31–1.94)
12 or more visits to any health practitioner	2.03 (1.78–2.31)	1.83 (1.60–2.10)
24 or more visits to any health practitioner^§^	2.64 (2.17–3.20)	2.29 (1.87–2.80)

CI = confidence interval.

* The raw data underlying the adjusted odds ratios are included in the Supporting Information, table 2.

† Adjusted for age group, and sex.

‡ Adjusted for age group, sex, socio‐economic status, financial hardship (childhood and current), and geographic remoteness.

§ Includes people with twelve or more visits.

### Maltreatment during childhood, by type

After adjusting for all other types of maltreatment, as well as for socio‐demographic characteristics, financial hardship (childhood and current), and geographic remoteness, the odds of overnight hospital admission during the preceding twelve months were higher for participants who reported sexual abuse (aOR, 1.24; 95% CI, 1.03–1.50) or physical abuse (aOR, 1.21; 95% CI, 1.00–1.46) (Box 1). The relative likelihood of hospitalisation with a mental disorder (compared with people who reported no maltreatment) was highest for people who reported emotional abuse (aOR, 2.30; 95% CI, 1.05–5.06); the likelihood of hospitalisation because of suicide risk was also higher in this group, but not statistically significantly (aOR, 1.82; 95% CI, 0.79–4.19). Participants who reported neglect were more likely to have been hospitalised with stroke (aOR, 5.52; 95% CI, 1.40–21.5) or alcohol‐related problems during the preceding twelve months (aOR, 15.9; 95% CI, 1.00–179) (Supporting Information, table 3). The odds of six or more visits to general practitioners was highest for people who reported sexual abuse (aOR, 1.75; 95% CI, 1.33–2.32) (Supporting Information, table 4).

### Multiple types of maltreatment during childhood

The likelihood of visits to health care professionals generally increased with the number of maltreatment types reported, but the differences between strata (ie, two *v* one types, or three or more *v* two types) were often not statistically significant. For example, compared with respondents who reported no maltreatment, the odds of at least one consultation with a psychiatrist, psychologist, or mental health nurse, and of six or more visits to general practitioners in the past twelve months, were each greater for people who reported three or more types of maltreatment than for those who reported two types or one type, but the confidence intervals for the respective estimates overlapped (with the exception of consultations with psychologists). Participants who reported three or more types of maltreatment were more likely to report 24 or more consultations with health care professionals during the preceding twelve months (*v* no maltreatment: aOR, 3.29; 95% CI, 2.61–4.13) than respondents who reported two types (aOR, 2.02; 95% CI, 1.55–2.62) or one type (aOR, 1.68; 95% CI, 1.31–2.17) (Box 4, Box 5).

Box 4 Likelihood of overnight hospital admissions and repeated consultations with health care professionals during preceding twelve months, by number of maltreatment types reported (*v* no maltreatment reported)*

**Table:** 

	Adjusted odds ratio (95% CI)	
Health service use	Partial^†^	Full^‡^
Admitted overnight to hospital at least once		
One maltreatment type	1.23 (0.99–1.54)	1.22 (0.98–1.52)
Two maltreatment types	1.55 (1.22–1.96)	1.43 (1.12–1.82)
Three to five maltreatment types	1.84 (1.50–2.24)	1.58 (1.26–1.96)
12 or more consultations with health care professionals		
One maltreatment type	1.41 (1.19–1.67)	1.38 (1.17–1.63)
Two maltreatment types	1.98 (1.65–2.37)	1.81 (1.50–2.18)
Three to five maltreatment types	2.89 (2.47–3.39)	2.53 (2.13–2.99)
24 or more consultations with health care professionals^§^		
One maltreatment type	1.73 (1.35–2.22)	1.68 (1.31–2.17)
Two maltreatment types	2.29 (1.78–2.95)	2.02 (1.55–2.62)
Three to five maltreatment types	3.91 (3.16–4.84)	3.29 (2.61–4.13)

CI = confidence interval.

* The raw data underlying the adjusted odds ratios, and for specific reasons for hospital admission and specific consultation types, are included in the Supporting Information, tables 5 and 6.

† Adjusted for age group, and sex.

‡ Adjusted for age group, sex, socio‐economic status, financial hardship (childhood and current), and geographic remoteness.

§ Includes people with twelve or more visits.

Box 5 Likelihood of overnight hospital admissions and consultations with health care professionals during preceding twelve months for participants who reported three to five types of maltreatment (*v* no maltreatment reported)*

**Table:** 

	Adjusted odds ratio (95% CI)	
Health service use	Partial^†^	Full^‡^
Admitted overnight to hospital at least once	1.84 (1.50–2.24)	1.58 (1.26–1.96)
Reasons for overnight hospital admissions		
Any mental disorder^§^	4.30 (1.80–10.4)	3.71 (1.50–9.19)
Anxiety	0.81 (0.28–2.34)	0.89 (0.34–2.34)
Depression	4.39 (0.70–27.6)	4.19 (0.60–30.3)
Drug‐related problems	2.67 (0.40–16.4)	1.22 (0.17–8.81)
Alcohol‐related problems	0.51 (0.12–2.10)	1.40 (0.30–6.62)
Suicide risk	5.22 (1.10–25.7)	2.15 (0.81–5.75)
Heart disease^¶^	2.27 (1.05–4.90)	1.28 (0.53–3.11)
Injury or results of injury	1.09 (0.68–1.76)	1.03 (0.62–1.72)
Asthma or chronic bronchitis	0.93 (0.23–3.83)	0.74 (0.21–2.67)
Diabetes	1.12 (0.21–5.99)	0.30 (0.04–2.44)
Cancer	1.18 (0.47–2.97)	1.38 (0.53–3.56)
Stroke	1.21 (0.41–3.62)	0.72 (0.21–2.54)
Consultations with health care professionals in the past twelve months		
At least one visit to psychologist	3.93 (3.23–4.78)	3.49 (2.83–4.31)
At least one visit to psychiatrist	4.55 (3.33–6.21)	3.95 (2.87–5.44)
At least one visit to mental health nurse	4.53 (2.95–6.96)	3.60 (2.28–5.69)
At least one visit to any medical specialist	1.51 (1.29–1.76)	1.45 (1.22–1.71)
1–5 visits to general practitioners	1.38 (1.06–1.79)	1.48 (1.11–1.98)
6 or more visits to general practitioners	3.68 (2.75–4.91)	3.27 (2.37–4.52)
1–5 visits to allied health professionals	1.62 (1.33–1.96)	1.53 (1.24–1.90)
6 or more visits to allied health professionals	2.36 (1.90–2.92)	2.14 (1.69–2.71)

CI = confidence interval.

* The raw data underlying the adjusted odds ratios are included in the Supporting Information, tables 5 and 6.

† Adjusted for age group, and sex.

‡ Adjusted for age group, sex, socio‐economic status, financial hardship (childhood and current), and geographic remoteness.

§ Includes schizophrenia, anxiety, depression, eating disorders, bipolar disorder, personality disorders, and (from the “Other” category) mental health, post‐traumatic stress disorder, and panic attack.

¶ Includes heart attack and (from the “Other” category) heart disease, heart disease/anaemia, heart disease/pneumonia, heart disease/surgery, heart failure/arrhythmia, heart failure, heart problem, heart surgery, surgery/heart problem.

### Differences in odds ratios between partially and fully adjusted models

After adjusting for geographic remoteness and socio‐economic status in the fully adjusted model, the relative odds for people maltreated during childhood reporting any overnight hospitalisation during the preceding twelve months with depression (3.3; 95% CI, 0.5–20 *v* 3.6; 95% CI, 0.6–21) or any mental disorder (2.4; 95% CI, 1.03–5.6 *v* 2.7; 95% CI, 1.1–6.5) were moderately lower than in the partially adjusted models; the adjusted odds ratio for suicide risk was much lower (1.4; 95% CI, 0.6–3.3 *v* 3.8; 95% CI, 0.8–18) (Box 2).

For six or more general practitioner consultations, the adjusted odds ratios were similarly smaller in the fully adjusted model (2.37; 95% CI, 1.87–3.02 *v* 2.66; 95% CI, 2.12–3.35), but that for 1–5 visits to general practitioners was moderately larger (1.37; 95% CI, 1.12–1.67 *v* 1.33; 95% CI, 1.10–1.61) (Box 3). The likelihood of hospital admission and high numbers of health care consultations for participants who reported three or more types of maltreatment were also moderately smaller after full adjustment, except for overnight hospital admission because of suicide risk; the adjusted odds ratio was larger and statistically significant in the partially adjusted model (5.22; 95% CI, 1.10–25.7) but not significant after full adjustment (2.15; 95% CI, 0.81–5.75) (Box 5).

## Discussion

We report the first investigation of associations between child maltreatment and health service use for a representative sample of Australians aged 16 years or more. ACMS data enabled us to disaggregate outcomes by type of maltreatment, and by number of maltreatment types, and to control for several factors. We found that child maltreatment was associated with greater likelihood of hospital admission with mental disorders and frequent consultations with health care professionals. These findings are consistent with those of overseas studies,^3^, ^4^, ^5^ and with other reports that child maltreatment is associated with increased health service use.^7^, ^8^, ^9^, ^10^

The few studies that have examined relationships between specific child maltreatment types and later health service use have found increased health care use and costs only in relation to neglect and physical and sexual abuse.^7^, ^8^, ^9^, ^10^, ^11^ Our findings confirm those of these studies, which found that the likelihood of hospitalisation was higher after physical and sexual abuse, after adjusting for experience of other maltreatment types. In addition, our study builds on other reports^9^, ^10^ with our findings of increased likelihood of hospital admission with mental health problems and of consultations with health professionals for people who report multiple types of maltreatment during childhood. The odds of 24 or more consultations with health care professionals during the preceding twelve months were greatest for people who had experienced three or more forms of child maltreatment.

Child maltreatment was not associated with greater likelihood of hospital admission for physical health reasons. This surprising finding might be explained by the fact that we did not analyse health service use by age group, and physical disorders would have been less prevalent in our sample because of our oversampling of people aged 16–24 years. Further, many conditions can be managed in primary care (eg, asthma, diabetes), reducing the need for hospitalisation.^25^ The COVID‐19 pandemic may also have altered health service use patterns. However, child maltreatment was associated with greater likelihood of large numbers of health care visits during the preceding twelve months, including to general practitioners and specialists, conceivably for both mental and physical health reasons. To complement our analysis, focused on health service use, it will be important that future ACMS analyses explore the relationship between child maltreatment and later physical health outcomes.

Child maltreatment was not associated with increased likelihood of hospital admission with injuries. One reason is that the sample did not include children under 16 years of age. Physical injury caused by maltreatment is common, especially during early childhood. A study of children's hospital admissions in Western Australia during 1980–2005 found that 97% of maltreatment‐related admissions involved injuries, predominantly in children aged 0–6 years.^26^

Child maltreatment can increase health service use via several intersecting pathways. Effects on brain development and epigenetic and neurobiological changes increase lifelong risk for physical and mental disorders; moreover, coping mechanisms, such as smoking and alcohol and drug use, can lead to injury and disease.^27^, ^28^, ^29^, ^30^, ^31^ Mental disorders and substance use during adolescence can also influence the impact of maltreatment history on health service use in adulthood. The ACMS found associations between child maltreatment and mental disorders that emerged during early adulthood.^17^ A prospective cohort study in the United States, with data collected at four time points, similarly found that the relationship between child maltreatment and increased health service use was partly explained by the increased risk of developing mental disorders; maltreated people with major depression or who used drugs during young adulthood (approximate age at follow‐up, 29 years) were significantly more likely to use general medical services in middle adulthood (approximate age at follow‐up, 41 years).^9^

Defining the contribution of child maltreatment to poor health and increased health service use is challenging, given the likely contribution of other factors, including socio‐economic disadvantage, place of residence and access to health services. Access to health services is also influenced by socio‐economic status.^32^ In addition, Australians living in rural and remote areas have lower life expectancy, poorer health outcomes, higher disease and injury burdens, and less access to and use of health services.^33^ Poorer health outcomes in rural and remote areas may also be related to poor nutrition, smoking and alcohol use, education and employment disadvantage,^33^ and higher rates of violence; people in remote areas are 24 times as likely to be hospitalised because of domestic violence than residents of major cities.^34^

Applying two separate levels of model adjustment allowed insights into the contributions of geographic remoteness and socio‐economic status as potential confounders affecting the relationship between maltreatment and health service use. We found that although most associations remained statistically significant after full adjustment, the odds ratios were generally smaller after full than partial adjustment, suggesting that remoteness and financial security influence these complex relationships.

Residual and unmeasured confounding may have affected our findings. Interactions between genetic and environmental factors influence the predisposition to mental disorders.^35^, ^36^ Child maltreatment may also be a marker of other household dysfunction that influences health outcomes and subsequent health service use. A recent systematic review found that the risks of health risk behaviours, mental disorders, substance use, self‐directed violence, and non‐communicable diseases were greater for people who reported four or more adverse childhood experiences, including maltreatment but also household mental illness, parental divorce, household criminality, and bullying.^37^ Further research is needed to understand the role of mediating and moderating variables and to ensure adequate adjustment of analyses for lifetime confounders. Future ACMS analyses will explore these relationships after adjusting for factors such as parental mental health and substance use, and peer bullying.

Similarly, protective factors — resilience, supportive family environments, safe schools and neighbourhoods — reduce the risks of the long term consequences of maltreatment.^31^, ^38^ Early, targeted interventions are vital, not only for supporting children directly, but also for their longer term wellbeing and reducing health system use. Integrated physical and psychological support and health care is needed to reduce the morbidity caused by maltreatment and to improve long term health.

### Limitations

Despite our large sample, small cell sizes for some reasons for hospital admission mean that some results should be interpreted with caution. Recall bias is possible, as some participants were asked to report on long passed events; however, moderate to good consistency of reports over time and under‐ rather than overreporting of maltreatment has been described.^39^ Participants with poor health may be more likely to disclose maltreatment,^40^ but we assessed maltreatment before health in the questionnaire to limit this bias. Measurement bias and questionable reliability of self‐reported health service use may also affect our findings.

The health service use items we used and methodology were based on those of the 2007 Australian National Survey of Mental Health and Wellbeing.^21^ We did not use the diagnostic criteria of the International Statistical Classification of Diseases (ICD‐10) or the Diagnostic and Statistical Manual of Mental Disorders (DSM‐5) for the hospitalisation health reasons because many participants would be unable to report at this formal level; for example, they may not know whether an admission was for ischaemic or hypertensive heart disease, but could report that it was for “heart disease”. Self‐report of health service use assumed participants knew enough to be able to report reasons for hospital admission from a list of nine major mental health and eleven physical health conditions (including “other”). Finally, the COVID‐19 pandemic may have influenced patterns of health service use, particularly hospital admissions, during the data collection period. A systematic review found that health care use declined around the world by about one‐third during the COVID‐19 pandemic.^41^

### Conclusion

The ACMS provides evidence that child maltreatment increases health service use later in life, particularly for people who have experienced multiple types of maltreatment. Better prevention strategies and targeted support for children and families at risk are needed. In addition, a more nuanced understanding of how the complex interactions between child maltreatment and individual and environmental protective factors affect health and wellbeing across life is needed to design effective strategies for improving long term outcomes. Our findings also enable timely quantification of the substantial lifetime economic and social costs of child maltreatment in Australia.

## Open access

Open access publishing facilitated by Queensland University of Technology, as part of the Wiley – Queensland University of Technology agreement via the Council of Australian University Librarians.

## Agency roles

The NHMRC funded the ACMS. The Australian government provided supplementary funding for several specific questions. The researchers were independent of the funders.

## Competing interests

No relevant disclosures.

## Data sharing statement

Under the registered data management plan, final datasets will be stored on the Australian Data Archive; access details will be published on the ACMS website (www.australianchildmaltreatmentstudy.org) in 2024. Under a multi‐institutional agreement, the survey instrument is the intellectual property of the research team. It will be made available under a Creative Commons licence after an embargo period.

## Supporting information


Supporting Information.
