# Supplementary material for: Child maltreatment and health service use: findings of the Australian Child Maltreatment Study
Source: Med J Aust. 2023 Apr 2;218(Suppl 6):S40–6. doi: 10.5694/mja2.51892 (PMC10952869; doi:10.5694/mja2.51892)
Supplement: Supplementary file 1 — Supporting Information. [file MJA2-218-S40-s001.pdf]

Supporting Information

Supplementary results

This appendix was part of the submitted manuscript and has been peer reviewed.
It is posted as supplied by the authors.

Appendix to: Pacella R, Nation A, Mathews B, et al. Child maltreatment and health service use: findings of the Australian Child Maltreatment Study. *Med J Aust* 2023; doi: 10.5694/mja2.51892.

Table 1. Numbers and unweighted proportions§ of survey respondents admitted to hospital during the preceding twelve months, by reported experience of child maltreatment

	Experienced any child maltreatment		
**Hospital admissions**	**No**	**Yes**	**All respondents**
**Total number of respondents**	3223	5280	8503
**Admitted overnight to hospital in the past 12 months**			
No	2827 (87.7%)	4397 (83.3%)	7224 (85.0%)
Yes (admitted one or more times)	361 (11.2%)	857 (16.2%)	1218 (14.3%)
Not stated	35 (1.1%)	26 (0.5%)	61 (0.7%)
**Reason for hospital admission in the past 12 months**			
Any mental disorder*	9 (0.3%)	63 (1.2%)	72 (0.8%)
Psychosis (including schizophrenia)	0	8 (0.2%)	8 (0.1%)
Anxiety	6 (0.2%)	25 (0.5%)	31 (0.4%)
Depression	2 (0.1%)	22 (0.4%)	24 (0.3%)
Bipolar disorder	0	11 (0.2%)	11 (0.1%)
Eating disorder	1 (< 0.1%)	7 (0.1%)	8 (0.1%)
Personality disorder	0	5 (0.1%)	5 (0.1%)
Drug-related problems	2 (0.1%)	12 (0.2%)	14 (0.2%)
Alcohol-related problems	7 (0.2%)	15 (0.3%)	22 (0.3%)
Suicide risk	2 (0.1%)	50 (0.9%)	52 (0.6%)
Heart disease combined†	16 (0.5%)	38 (0.7%)	54 (0.6%)
Heart attack	9 (0.3%)	15 (0.3%)	24 (0.3%)
Injury or results of injury	74 (2.3%)	177 (3.4%)	251 (3.0%)
Asthma or chronic bronchitis	5 (0.2%)	16 (0.3%)	21 (0.2%)
Diabetes	3 (0.1%)	8 (0.2%)	11 (0.1%)
Arthritis	2 (0.1%)	2 (< 0.1%)	4 (< 0.1%)
Cancer	11 (0.3%)	26 (0.5%)	37 (0.4%)
Stroke	8 (0.2%)	13 (0.2%)	21 (0.2%)
Stomach or duodenal ulcer	7 (0.2%)	22 (0.4%)	29 (0.3%)
Gall bladder or liver trouble	10 (0.3%)	29 (0.5%)	39 (0.5%)
Complications of pregnancy or childbirth	1 (< 0.1%)	11 (0.2%)	12 (0.1%)
Coronavirus	0	8 (0.2%)	8 (0.1%)
Other	227 (7.0%)	493 (9.3%)	720 (8.5%)

§ Note that unweighted proportions are presented here and ACMS uses population weights to estimate population proportions. The ACMS team can be contacted for weighted proportions.

* Includes schizophrenia, anxiety, depression, eating disorders, bipolar disorder, personality disorders, and (from the "Other" category) mental health reason, post-traumatic stress disorder and panic attack.

† Includes heart attack and (from the "Other" category) heart disease, heart disease/anaemia, heart disease/pneumonia, heart disease/surgery, heart failure/arrhythmia, heart failure, heart problem, heart surgery, surgery/heart problem.

Table 2. Numbers and unweighted proportions[§] of survey respondents visiting health care professionals during the preceding twelve months, by reported experience of child maltreatment

	Experienced any child maltreatment		
**Consultation type**	**No**	**Yes**	**All respondents**
At least one visit to psychologist			
Not stated	31 (1.0%)	36 (0.7%)	67 (0.8%)
No	2816 (87.4%)	3873 (73.4%)	6689 (78.7%)
Yes	376 (11.7%)	1371 (26.0%)	1747 (20.5%)
At least one visit to psychiatrist			
Not stated	36 (1.1%)	48 (0.9%)	84 (1.0%)
No	3084 (95.7%)	4709 (89.2%)	7793 (91.7%)
Yes	103 (3.2%)	523 (9.9%)	626 (7.4%)
At least one visit to mental health nurse			
Not stated	29 (0.9%)	50 (0.9%)	79 (0.9%)
No	3145 (97.6%)	4910 (93.0%)	8055 (94.7%)
Yes	49 (1.5%)	320 (6.1%)	369 (4.3%)
Visits to medical specialist			
Not stated	23 (0.7%)	29 (0.5%)	52 (0.6%)
No	2262 (70.2%)	3497 (66.2%)	5759 (67.7%)
Yes	938 (29.1%)	1754 (33.2%)	2692 (31.7%)
Visits to general practitioner			
Not stated	36 (1.1%)	50 (0.9%)	86 (1.0%)
0	439 (13.6%)	498 (9.4%)	937 (11.0%)
1–5 visits	2130 (66.1%)	3077 (58.3%)	5207 (61.2%)
6 or more visits	618 (19.2%)	1655 (31.3%)	2273 (26.7%)
Visits to any allied health professional			
Not stated	30 (0.9%)	50 (0.9%)	80 (0.9%)
0	2409 (74.7%)	3503 (66.3%)	5912 (69.5%)
1–5 visits	483 (15.0%)	1004 (19.0%)	1487 (17.5%)
6 or more visits	301 (9.3%)	723 (13.7%)	1024 (12.0%)
Visits to complementary therapist			
Not stated	27 (0.8%)	36 (0.7%)	63 (0.7%)
0	2836 (88.0%)	4339 (82.2%)	7175 (84.4%)
1–5 visits	221 (6.9%)	565 (10.7%)	786 (9.2%)
6 or more visits	139 (4.3%)	340 (6.4%)	479 (5.6%)
Visits to any health practitioner			
11 visits or less	2592 (80.4%)	3456 (65.5%)	6048 (71.1%)
12–23 visits	398 (12.4%)	884 (16.7%)	1282 (15.1%)
24 or more visits	233 (7.2%)	940 (17.8%)	1173 (13.8%)

[§] Note that unweighted proportions are presented here and ACMS uses population weights to estimate population proportions. The ACMS team can be contacted for weighted proportions.

Table 3. Likelihood of reasons for overnight hospital admission during the preceding twelve months (maltreatment reported *v* not reported), by child maltreatment type

		Adjusted odds ratio (95% CI)	
**Reason for admission**	**Admissions**	**Partial***	**Full†**
Any mental disorder‡	72		
No maltreatment reported	9	1	1
Emotional abuse	52	2.33 (1.08–5.06)	2.30 (1.05–5.06)
Neglect	23	1.76 (0.83–3.74)	1.61 (0.70–3.71)
Physical abuse	40	1.36 (0.59–3.12)	1.31 (0.60–2.85)
Sexual abuse	40	1.49 (0.72–3.06)	1.39 (0.68–2.83)
Exposure to domestic violence	47	0.88 (0.38–2.03)	0.89 (0.36–2.18)
Anxiety	31		
No maltreatment reported	6	1	1
Emotional abuse	19	0.86 (0.30–2.46)	0.90 (0.29–2.85)
Neglect	9	2.91 (1.03–8.21)	2.66 (0.81–8.70)
Physical abuse	15	0.52 (0.20–1.35)	0.51 (0.23–1.13)
Sexual abuse	17	1.23 (0.46–3.28)	1.29 (0.53–3.13)
Exposure to domestic violence	21	1.52 (0.45–5.17)	2.02 (0.44–9.20)
Depression	24		
No maltreatment reported	2	1	1
Emotional abuse	18	1.08 (0.30–3.98)	1.21 (0.30–4.83)
Neglect	8	1.82 (0.60–5.48)	1.55 (0.50–4.78)
Physical abuse	16	1.26 (0.40–3.95)	1.25 (0.41–3.79)
Sexual abuse	16	1.76 (0.47–6.57)	1.75 (0.48–6.34)
Exposure to domestic violence	18	2.16 (0.50–9.22)	2.22 (0.40–13.4)
Drug-related problems	17		
No maltreatment reported	2	1	1
Emotional abuse	8	0.54 (0.06–5.27)	0.61 (0.09–4.02)
Neglect	4	1.19 (0.29–4.91)	1.04 (0.14–7.98)
Physical abuse	9	2.46 (0.20–24.9)	1.94 (0.40–10.6)
Sexual abuse	7	0.51 (0.12–2.14)	0.41 (0.08–2.07)
Exposure to domestic violence	10	3.71 (0.80–17.1)	2.21 (0.50–9.84)
Alcohol-related problems	22		
No maltreatment reported	7	1	1
Emotional abuse	8	1.09 (0.15–7.88)	0.93 (0.21–4.06)
Neglect	6	8.63 (1.30–56.1)	15.9 (1.00 – 179)
Physical abuse	10	1.03 (0.36–2.90)	1.35 (0.45–4.03)
Sexual abuse	8	1.07 (0.29–3.98)	1.64 (0.56–4.85)
Exposure to domestic violence	10	0.20 (0.05–0.76)	0.18 (0.03–0.93)
Suicide risk	52		
No maltreatment reported	2	1	1
Emotional abuse	41	2.62 (0.98–7.00)	1.82 (0.79–4.19)
Neglect	13	0.84 (0.36–1.96)	1.17 (0.52–2.62)
Physical abuse	38	2.07 (0.74–5.76)	1.42 (0.56–3.58)
Sexual abuse	34	1.20 (0.53–2.74)	1.17 (0.55–2.48)
Exposure to domestic violence	36	0.85 (0.40–1.81)	1.02 (0.47–2.26)

(continues)

Table 3. (continued)

	Adjusted odds ratio (95% CI)		
**Reason for admission**	**Admissions**	**Partial***	**Full†**
Heart disease combined§	54		
No maltreatment reported	16	1	1
Emotional abuse	22	0.89 (0.43–1.83)	0.70 (0.32–1.50)
Neglect	6	0.56 (0.18–1.74)	0.29 (0.07–1.22)
Physical abuse	31	3.52 (1.76–7.01)	3.39 (1.71–6.73)
Sexual abuse	16	0.83 (0.38–1.83)	0.71 (0.33–1.51)
Exposure to domestic violence	25	1.07 (0.58–1.97)	0.91 (0.50–1.69)
Injury or results of injury	251		
No maltreatment reported	74	1	1
Emotional abuse	97	0.81 (0.50–1.30)	0.82 (0.51–1.31)
Neglect	25	0.76 (0.39–1.48)	0.67 (0.33–1.39)
Physical abuse	101	1.15 (0.75–1.79)	1.10 (0.70–1.73)
Sexual abuse	84	1.03 (0.67–1.59)	1.02 (0.66–1.58)
Exposure to domestic violence	124	1.23 (0.82–1.84)	1.24 (0.81–1.90)
Asthma or chronic bronchitis	21		
No maltreatment reported	5	1	1
Emotional abuse	8	0.16 (0.03–0.78)	0.17 (0.03–0.89)
Neglect	5	2.04 (0.44–9.35)	2.28 (0.50–11.6)
Physical abuse	9	2.57 (0.60–10.9)	2.61 (0.50–13.4)
Sexual abuse	11	1.05 (0.32–3.48)	0.87 (0.25–3.06)
Exposure to domestic violence	11	2.27 (0.54–9.49)	1.84 (0.62–5.43)
Diabetes	11		
No maltreatment reported	3	1	1
Emotional abuse	7	0.94 (0.10–10.1)	0.77 (0.10–5.89)
Neglect	3	1.44 (0.26–7.90)	1.39 (0.20–10.6)
Physical abuse	5	1.38 (0.20–10.8)	1.23 (0.10–11.0)
Sexual abuse	3	0.43 (0.10–1.75)	0.39 (0.07–2.29)
Exposure to domestic violence	6	2.98 (0.60–14.6)	1.49 (0.33–6.65)
Cancer	37		
No maltreatment reported	11	1	1
Emotional abuse	16	0.90 (0.41–1.98)	0.99 (0.43–2.29)
Neglect	6	1.94 (0.55–6.89)	1.44 (0.39–5.37)
Physical abuse	16	0.73 (0.33–1.64)	0.78 (0.33–1.88)
Sexual abuse	14	0.76 (0.31–1.86)	0.74 (0.33–1.69)
Exposure to domestic violence	18	1.52 (0.61–3.80)	1.72 (0.55–5.33)
Stroke	21		
No maltreatment reported	8	1	1
Emotional abuse	8	0.66 (0.21–2.10)	0.52 (0.12–2.36)
Neglect	5	3.72 (1.10–12.9)	5.52 (1.40–21.5)
Physical abuse	9	0.97 (0.31–3.02)	0.85 (0.25–2.92)
Sexual abuse	6	0.63 (0.25–1.57)	0.54 (0.19–1.52)
Exposure to domestic violence	12	1.87 (0.76–4.56)	1.73 (0.68–4.45)

CI = confidence interval.

* Adjusted for other maltreatment types, age group, and sex.

† Adjusted for other maltreatment types, age group, sex, socio-economic status, financial hardship (childhood and current), and geographic remoteness.

‡ Includes schizophrenia, anxiety, depression, eating disorders, bipolar disorder, personality disorders, and (from the "Other" category) mental health, post-traumatic stress disorder, and panic attack.

§ Includes heart attack and (from the "Other" category) heart disease, heart disease/anaemia, heart disease/pneumonia, heart disease/surgery, heart failure/arrhythmia, heart failure, heart problem, heart surgery, surgery/heart problem.

Table 4. Likelihood of consultations with health care professionals during preceding twelve months (maltreatment reported *v* not reported), by child maltreatment type

		Adjusted odds ratio (95% CI)	
**Consultation type**	**Number**	**Partial***	**Full†**
At least one visit to psychologist	1747		
No maltreatment reported	376	1	1
Emotional abuse	921	1.95 (1.61–2.36)	1.92 (1.58–2.32)
Neglect	286	1.24 (0.98–1.56)	1.18 (0.92–1.52)
Physical abuse	750	1.22 (1.01–1.47)	1.20 (0.99–1.45)
Sexual abuse	744	1.65 (1.40–1.95)	1.60 (1.36–1.90)
Exposure to domestic violence	963	1.17 (0.99–1.40)	1.15 (0.96–1.38)
At least one visit to psychiatrist	626		
No maltreatment reported	103	1	1
Emotional abuse	347	1.46 (1.08–1.98)	1.44 (1.06–1.95)
Neglect	120	1.25 (0.91–1.72)	1.16 (0.83–1.62)
Physical abuse	310	1.65 (1.26–2.16)	1.59 (1.21–2.08)
Sexual abuse	293	1.58 (1.23–2.03)	1.49 (1.16–1.92)
Exposure to domestic violence	358	1.19 (0.90–1.58)	1.19 (0.90–1.57)
At least one visit to mental health nurse	369		
No maltreatment reported	49	1	1
Emotional abuse	221	1.64 (1.10–2.45)	1.61 (1.07–2.41)
Neglect	78	1.33 (0.89–1.99)	1.19 (0.77–1.82)
Physical abuse	189	1.16 (0.80–1.68)	1.08 (0.75–1.56)
Sexual abuse	195	1.92 (1.39–2.66)	1.72 (1.23–2.39)
Exposure to domestic violence	236	1.32 (0.94–1.86)	1.30 (0.91–1.85)
At least one visit to any medical specialist	2692		
No maltreatment reported	938	1	1
Emotional abuse	920	1.00 (0.85–1.17)	0.99 (0.85–1.16)
Neglect	254	0.91 (0.73–1.15)	0.89 (0.70–1.13)
Physical abuse	912	1.20 (1.04–1.39)	1.19 (1.02–1.37)
Sexual abuse	876	1.33 (1.15–1.53)	1.31 (1.14–1.52)
Exposure to domestic violence	1130	1.11 (0.96–1.27)	1.09 (0.94–1.25)
1–5 visits to general practitioners	5207		
No maltreatment reported	2130	1	1
Emotional abuse	1494	1.06 (0.81–1.38)	1.06 (0.81–1.38)
Neglect	363	0.72 (0.47–1.09)	0.79 (0.51–1.23)
Physical abuse	1461	1.00 (0.79–1.27)	1.03 (0.81–1.31)
Sexual abuse	1282	1.17 (0.91–1.50)	1.23 (0.96–1.58)
Exposure to domestic violence	2007	1.48 (1.17–1.86)	1.48 (1.17–1.86)
6 or more visits to general practitioners	2273		
No maltreatment reported	618	1	1
Emotional abuse	983	1.47 (1.10–1.96)	1.42 (1.06–1.91)
Neglect	319	0.90 (0.58–1.40)	0.89 (0.56–1.41)
Physical abuse	893	1.27 (0.98–1.66)	1.28 (0.97–1.67)
Sexual abuse	883	1.82 (1.38–2.40)	1.75 (1.33–2.32)
Exposure to domestic violence	1110	1.75 (1.36–2.26)	1.69 (1.30–2.18)

(continues)

Table 4. (continued)

	Adjusted odds ratio (95% CI)		
**Consultation type**	**Number**	**Partial***	**Full†**
1–5 visits to allied health professionals	1487		
No maltreatment reported	483	1	1
Emotional abuse	554	1.18 (0.97–1.43)	1.18 (0.97–1.43)
Neglect	155	1.17 (0.89–1.53)	1.14 (0.85–1.53)
Physical abuse	504	1.16 (0.97–1.40)	1.15 (0.96–1.38)
Sexual abuse	474	1.22 (1.02–1.45)	1.19 (1.00–1.43)
Exposure to domestic violence	654	1.00 (0.84–1.19)	0.99 (0.83–1.18)
6 or more visits to allied health professionals	1024		
No maltreatment reported	301	1	1
Emotional abuse	416	1.19 (0.96–1.49)	1.18 (0.94–1.48)
Neglect	152	1.58 (1.18–2.11)	1.55 (1.15–2.09)
Physical abuse	401	1.19 (0.97–1.47)	1.18 (0.96–1.46)
Sexual abuse	389	1.45 (1.19–1.76)	1.43 (1.17–1.75)
Exposure to domestic violence	487	1.14 (0.93–1.39)	1.12 (0.91–1.37)

CI = confidence interval.

* Adjusted for other maltreatment types, age group, and sex.

† Adjusted for other maltreatment types, age group, sex, socio-economic status, financial hardship (childhood and current), and geographic remoteness.

Table 5. Likelihood for overnight hospital admissions during the preceding twelve months (maltreatment reported *v* not reported), by number of maltreatment types reported

Hospital admissions	Maltreatment types reported	Number	Adjusted odds ratio (95% CI)	
			Partial*	Full†
Admitted overnight to hospital at least once	None	361	1	1
	1 type	257	1.23 (0.99–1.54)	1.22 (0.98–1.52)
	2 types	204	1.55 (1.22–1.96)	1.43 (1.12–1.82)
	3–5 types	396	1.84 (1.50–2.24)	1.58 (1.26–1.96)
Reasons for overnight hospital admission				
Any mental disorder‡	None	9	1	1
	1 type	4	1.36 (0.34–5.43)	1.39 (0.36–5.44)
	2 types	17	2.41 (0.86–6.76)	2.16 (0.79–5.92)
	3–5 types	42	4.30 (1.80–10.4)	3.71 (1.50–9.19)
Anxiety	None	6	1	1
	1 type	1	0.56 (0.07–4.83)	0.61 (0.07–5.41)
	2 types	9	1.03 (0.29–3.64)	1.20 (0.43–3.37)
	3–5 types	15	0.81 (0.28–2.34)	0.89 (0.34–2.34)
Depression	None	2	1	1
	1 type	1	2.12 (0.20–26.4)	2.18 (0.20–31.0)
	2 types	6	4.16 (0.60–28.3)	3.52 (0.50–23.9)
	3–5 types	15	4.39 (0.70–27.6)	4.19 (0.60–30.3)
Drug-related problems	None	2	1	1
	1 type	1	0.33 (0.03–4.09)	0.28 (0.02–3.19)
	2 types	3	9.37 (1.40–62.9)	6.36 (1.10–37.9)
	3–5 types	8	2.67 (0.40–16.4)	1.22 (0.17–8.81)
Alcohol-related problems	None	7	1	1
	1 type	3	0.21 (0.04–1.02)	0.34 (0.06–1.80)
	2 types	6	1.36 (0.30–6.30)	1.97 (0.45–8.62)
	3–5 types	6	0.51 (0.12–2.10)	1.40 (0.30–6.62)
Suicide risk	None	2	1	1
	1 type	5	1.04 (0.16–6.79)	0.66 (0.22–1.96)
	2 types	10	4.23 (0.70–24.5)	1.60 (0.50–5.06)
	3–5 types	35	5.22 (1.10–25.7)	2.15 (0.81–5.75)
Heart disease combined§	None	16	1	1
	1 type	9	1.33 (0.53–3.35)	1.17 (0.48–2.87)
	2 types	10	1.72 (0.65–4.54)	1.31 (0.48–3.58)
	3–5 types	19	2.27 (1.05–4.90)	1.28 (0.53–3.11)
Injury or results of injury	None	74	1	1
	1 type	60	1.19 (0.71–1.98)	1.17 (0.70–1.97)
	2 types	36	0.99 (0.55–1.76)	0.93 (0.51–1.69)
	3–5 types	81	1.09 (0.68–1.76)	1.03 (0.62–1.72)
Asthma or chronic bronchitis	None	5	1	1
	1 type	6	0.41 (0.11–1.60)	0.45 (0.11–1.95)
	2 types	2	1.49 (0.20–10.0)	1.37 (0.21–8.77)
	3–5 types	8	0.93 (0.23–3.83)	0.74 (0.21–2.67)

(continues)

Table 5. (continued)

Hospital admissions	Maltreatment types reported	Number	Adjusted odds ratio (95% CI)	
			Partial*	Full†
Diabetes	None	3	1	1
	1 type	1	0.11 (0.01–1.14)	0.04 (0.00–0.63)
	2 types	3	3.36 (0.50–21.7)	1.38 (0.24–8.05)
	3–5 types	4	1.12 (0.21–5.99)	0.30 (0.04–2.44)
Cancer	None	11	1	1
	1 type	8	1.19 (0.44–3.22)	1.25 (0.42–3.78)
	2 types	4	0.51 (0.15–1.82)	0.54 (0.14–1.99)
	3–5 types	14	1.18 (0.47–2.97)	1.38 (0.53–3.56)
Stroke	None	8	1	1
	1 type	2	0.14 (0.03–0.72)	0.09 (0.02–0.55)
	2 types	2	0.55 (0.10–2.96)	0.30 (0.05–1.79)
	3–5 types	9	1.21 (0.41–3.62)	0.72 (0.21–2.54)

CI = confidence interval.

* Adjusted for age group, and sex.

† Adjusted for age group, sex, socio-economic status, financial hardship (childhood and current), and geographic remoteness.

‡ Includes schizophrenia, anxiety, depression, eating disorders, bipolar disorder, personality disorders, and (from the "Other" category) mental health, post-traumatic stress disorder, and panic attack.

§ Includes heart attack and (from the "Other" category) heart disease, heart disease/anaemia, heart disease/pneumonia, heart disease/surgery, heart failure/arrhythmia, heart failure, heart problem, heart surgery, surgery/heart problem.

Table 6. Likelihood of consultations with health care professionals during preceding twelve months (maltreatment reported *v* not reported), by number of maltreatment types reported

Consultation type	Maltreatment types reported	Number	Adjusted odds ratio (95% CI)	
			Partial*	Full†
At least one visit to psychologist	None	376	1	1
	1 type	313	1.56 (1.24–1.95)	1.52 (1.21–1.91)
	2 types	339	2.72 (2.17–3.42)	2.46 (1.94–3.12)
	3–5 types	719	3.93 (3.23–4.78)	3.49 (2.83–4.31)
At least one visit to psychiatrist	None	103	1	1
	1 type	119	2.24 (1.56–3.21)	2.21 (1.54–3.17)
	2 types	124	3.32 (2.32–4.74)	3.02 (2.10–4.36)
	3–5 types	280	4.55 (3.33–6.21)	3.95 (2.87–5.44)
At least one visit to mental health nurse	None	49	1	1
	1 type	57	1.85 (1.09–3.14)	1.83 (1.07–3.11)
	2 types	75	3.06 (1.89–4.95)	2.62 (1.59–4.30)
	3–5 types	188	4.53 (2.95–6.96)	3.60 (2.28–5.69)
At least one visit to any medical specialist	None	938	1	1
	1 type	601	1.13 (0.96–1.33)	1.11 (0.95–1.31)
	2 types	434	1.22 (1.02–1.46)	1.17 (0.97–1.41)
	3–5 types	719	1.51(1.29–1.76)	1.45 (1.22–1.71)
1–5 visits to general practitioners	None	2130	1	1
	1 type	1198	1.21 (0.95–1.55)	1.22 (0.95–1.55)
	2 types	808	1.47 (1.09–1.97)	1.51 (1.12–2.03)
	3–5 types	1071	1.38 (1.06–1.79)	1.48 (1.11–1.98)
6 or more visits to general practitioners	None	618	1	1
	1 type	486	1.81 (1.36–2.42)	1.73 (1.29–2.32)
	2 types	394	2.76 (1.97–3.88)	2.48 (1.76–3.49)
	3–5 types	775	3.68 (2.75–4.91)	3.27 (2.37–4.52)
1–5 visits to allied health professionals	None	483	1	1
	1 type	331	1.24 (1.02–1.51)	1.23 (1.00–1.50)
	2 types	266	1.39 (1.11–1.72)	1.33 (1.06–1.67)
	3–5 types	407	1.62 (1.33–1.96)	1.53 (1.24–1.90)
6 or more visits to allied health professionals	None	301	1	1
	1 type	213	1.29 (1.02–1.65)	1.27 (1.00–1.62)
	2 types	161	1.60 (1.23–2.08)	1.49 (1.14–1.95)
	3–5 types	349	2.36 (1.90–2.92)	2.14 (1.69–2.71)

CI = confidence interval.

* Adjusted for age group, and sex.

† Adjusted for age group, sex, socio-economic status, financial hardship (childhood and current), and geographic remoteness.
